# Sudan red dye: a new agent causing type-2 occupational asthma

**DOI:** 10.1186/s13223-020-0404-8

**Published:** 2020-01-30

**Authors:** David Clofent, Miquel de Homdedeu, Mariana Muñoz-Esquerre, María Jesús Cruz, Xavier Muñoz

**Affiliations:** 1 Servicio de Neumologia, Hospital Universitario Vall d’Hebron, Passeig Vall d’Hebron 119, 08035 Barcelona, Spain; 2 CIBER Enfermedades Respiratorias (CibeRes), Madrid, Spain; 3 Servicio de Neumologia, Hospital Universitario de Bellvitge-IDIBELL, Universidad de Barcelona, Barcelona, Spain; 4 Departamento de Biología Celular, Fisiología e Inmunología, Universitat Autònoma de Barcelona, Barcelona, Spain

**Keywords:** Allergy, Specific inhalation challenge, Azoic agent

## Abstract

**Background:**

Sudan red or 1-[(2-methoxyphenyl)azo]-2-naphthol is a low molecular weight azoic agent widely used in industry, particularly in the production of hair dyes. The use of this product in the food industry is prohibited due to its potential carcinogenic effect, but no respiratory involvement has been reported to date.

**Case presentation:**

We present the case of a 46-year-old female patient who had been working in a cosmetics packaging company for 20 years. The patient developed occupational asthma to a red azo dye known as Sudan red. The diagnosis was confirmed by specific bronchial provocation test. Induced sputum samples were obtained previously and in the 24 h following the procedure, with a rise in the percentage of eosinophils from 10 to 65%.

**Conclusions:**

This report describes the case of a patient who developed OA caused by exposure to an azoic dye called Sudan red. The clinical and analytical features suggest a type 2-related asthma; however, we are not yet able to confirm the specific pathophysiological mechanism. The extensive use of azo dyes in industry means that it is particularly important to describe their implications for health, which are probably underestimated at present.

## Background

Occupational asthma (OA) is a work-related condition characterized by variable airflow obstruction and/or by bronchial hyperresponsiveness to the conditions of a specific working environment. Hundreds of agents are known developers of OA, among them more than 100 chemical entities, whose mechanism of action is often unknown [1, 2].

Various dyestuffs are considered to be causes of occupational respiratory diseases, which are mainly described in workers in textile and cosmetic industries [3–11]. Azoic dyes, which are synthesized from primary aromatic amines, are the most extensively used synthetic organic colorants worldwide [12]. Despite the widespread use of these products, there are few reports of their relationship to OA and it is likely that dyestuff-related OA is underdiagnosed.

Sudan red or 1-[(2-methoxyphenyl)azo]-2-naphthol is a low molecular weight azoic agent widely used in industry, particularly in the production of hair dyes. The use of this product in the food industry is prohibited due to its potential carcinogenic effect, but no respiratory involvement has been reported to date [13].

## Case presentation

We report the case of a 46-year-old female non-smoker without medical history of interest, who had been working in a cosmetics packaging company for 20 years. She had undergone daily exposure to several substances including a red azo dye known as Sudan red.

Over the past 15 years, the patient had experienced dyspnea, dry cough, occasional wheezing, facial edema, rhinitis and conjunctivitis. These symptoms had worsened in the last 3 years; she had frequently required emergency-room assistance for acute episodes of bronchospasm and had been admitted to hospital on four times. The patient’s symptoms responded to inhaled long acting beta-2 agonist and inhaled corticosteroids, and were clearly work-related since they improved on weekends, during vacations, and after she finally left her job.

Physical examination and chest radiograph were normal. Blood tests showed eosinophilia (500 cells/mm^3^) and increased total serum IgE (846 KU/L). Lung function study revealed an obstructive ventilatory pattern with a forced vital capacity (FVC) of 3.14 L (91%), a forced expiratory volume in one second (FEV1) of 2.12 L (76%) and FEV1/FVC of 67%. Methacholine challenge test was positive, with a PC20 of 0.85 mg/mL and a fractional exhaled nitric oxide test (FENO) of 47.2 ppb.

**Fig. 1:**
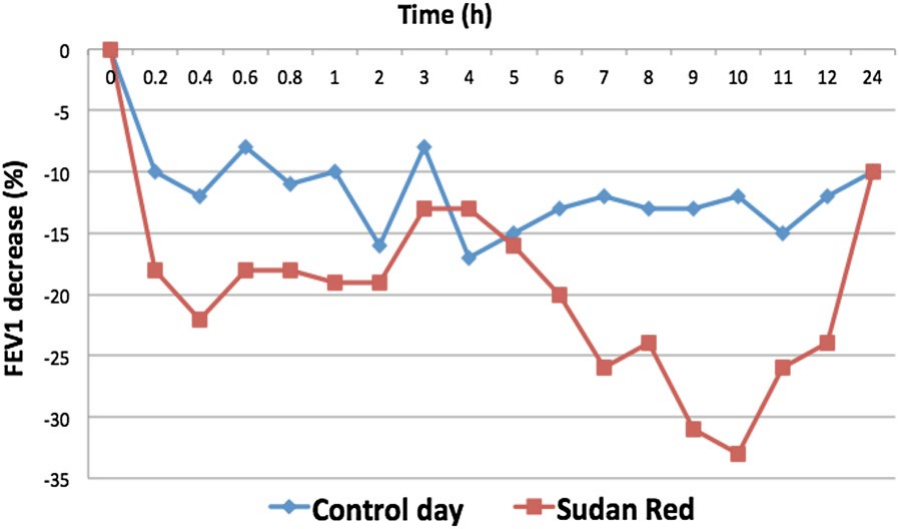
Result of specific inhalation challenge to Sudan red

With a suspected diagnosis of OA, the patient underwent a specific inhalation challenge (SIC) with the azoic dye, in accordance with the recommendations in the European Respiratory Society guidelines [14]. The patient was exposed to a mixture of 2 g of “Sudan Red” and 100 g of lactose powder, tipped from one tray to another 30 cm away from her face for 10 min; she continued treatment with inhaled long acting beta-2 agonist and inhaled corticosteroids due to the persistence of bronchospasm. During the procedure, she suffered dyspnea and cough, and experienced a dual positive response with a fall in FEV1 of 22% in the first 20 min and another of 33% approximately 10 h after the exposure. No significant changes in FEV1 were observed in response to a control challenge of lactose powder alone conducted on a separate day (Fig. 1).

Induced sputum samples were obtained previously and in the 24 h following the procedure, with a rise in the percentage of eosinophils from 10 to 65%. Methacholine and FENO tests did not present significant variations from the results obtained prior to the SIC. The diagnosis of OA due to Sudan Red was established, and the patient was advised to avoid the causal agent.

## Discussion

To our knowledge, this is the first reported case of OA caused by Sudan red dye. The increase of eosinophils in induced sputum is a particularly relevant finding, since it strongly suggests a type 2 mechanism of airway inflammation.

Since the first reports of dye-induced OA in 1978 by Alanko et al. [8], several cases have been published, most of them caused by exposure to reactive dyes in textile industries. In the early 1990s, on the basis of positive skin prick and RAST-inhibition tests in workers who developed allergic lower respiratory symptoms, authors such as Park HS [10], Docker [7] and Nilsson [3] proposed an IgE-mediated immunological mechanism. It was suggested that airborne dye molecules may act as haptens and induce IgE-mediated hypersensitivity reactions, However, none of these reports obtained cytological analysis in induced sputum.

Various pathophysiological pathways have been described in OA, depending on whether it is induced by a sensitizing or an irritant agent. While irritant-induced asthma is attributed to a non-immunological mechanism, sensitizing agents induce asthma through mechanisms which may or may not be type-2-dependent. In fact, a recent study showed that both high-molecular-weight agents (with an IgE-mediated mechanism) and low-molecular-weight agents (with as yet undefined mechanisms) may generate eosinophilic or neutrophilic responses [15].

In the present case report, the rise of eosinophils in induced sputum after the SIC suggests the involvement of a type-2 mechanism of airway inflammation. In addition, the presence of high levels of IgE and the dual positive response to the SIC may support an IgE-mediated mechanism and consequently a possible Th2 enrollment. However, the increase in eosinophils cannot rule out a response mediated by innate lymphocytic immunity (ILC2) or a combination of both.

## Conclusions

This report describes the case of a patient who developed OA caused by exposure to an azoic dye called Sudan red. The clinical and analytical features suggest a type 2-related asthma; however, we are not yet able to confirm the specific pathophysiological mechanism. The extensive use of azo dyes in industry means that it is particularly important to describe their implications for health, which are probably underestimated at present.

## Data Availability

Yes.

## Abbreviations

FENO: fractional exhaled nitric oxide test
FEV1: forced expiratory volume in one second
FVC: forced vital capacity
OA: occupational asthma
SIC: specific inhalation challenge
